# Supplementary material for: Molecular Modelling Hurdle in the Next-Generation Sequencing Era
Source: Int J Mol Sci. 2022 Jun 28;23(13):7176. doi: 10.3390/ijms23137176 (PMC9266691; doi:10.3390/ijms23137176)
Supplement: Supplementary file 1 [file ijms-23-07176-s001.zip › Table_S1.pdf]

Table S1. Enrichment Analysis Omim Disease.

Term	p-value	q-value
anemia	8.900845503514137e-17	8.010760953162724e-15
leukemia	2.97309780471867e-14	1.3378940121234013e-12
cardiomyopathy	3.9679132287527104e-11	1.1903739686258131e-09
myopathy	5.869720293780825e-11	1.3206870661006858e-09
diabetes	2.418530152306121e-10	4.3533542741510176e-09
diabetes mellitus, type 2	3.7290647082693876e-10	5.593597062404081e-09
blood	1.0307979930551578e-09	1.3253117053566316e-08
cardiomyopathy, hypertrophic	8.399106171743645e-09	9.4489944432116e-08
colorectal cancer	1.4984172874036155e-08	1.4050990228894967e-07
cardiomyopathy, dilated	1.5854979700517008e-08	1.4050990228894967e-07
immunodeficiency	1.7173432501982737e-08	1.4050990228894967e-07
retinitis pigmentosa	6.833749727172472e-08	5.125312295379353e-07
encephalopathy	2.2401907823160028e-07	1.5509013108341559e-06
charcot-marie-tooth disease	4.152255358614041e-07	2.6693070162518835e-06
cdeficiency	6.691882092992156e-07	3.5594729153421218e-06
glycogen storage disease	6.691882092992156e-07	3.5594729153421218e-06
disorder of glycosylation	6.723448840090675e-07	3.5594729153421218e-06
neuropathy	9.52571937174924e-07	4.76285968587462e-06
long qt syndrome	1.9987932960061957e-06	9.467968244239874e-06
bardet-biedl syndrome	2.310538172150581e-06	1.0397421774677615e-05
obesity	4.269142597110605e-06	1.829632541618831e-05
muscular dystrophy	4.720272733051294e-06	1.931020663520984e-05
cataract	5.4383211535268435e-06	2.1280387122496344e-05
gastric cancer	5.9695806779447125e-06	2.2349465837044675e-05
adenoma	6.456512352924017e-06	2.2349465837044675e-05
leigh syndrome	6.456512352924017e-06	2.2349465837044675e-05
diabetes mellitus	7.879635137093125e-06	2.5327398654942187e-05
epilepsy	7.879635137093125e-06	2.5327398654942187e-05
myocardial infarction	1.2721153115436082e-05	3.94794407030775e-05
asthma	1.4143757513532299e-05	4.2431272540596896e-05
epidermolysis bullosa	1.7954358546894954e-05	5.049663341314206e-05
leber amaurosis	1.7954358546894954e-05	5.049663341314206e-05
macular degeneration	1.9821694656651207e-05	5.4059167245412376e-05
lymphoma	3.333036583989179e-05	8.351427918110758e-05
parkinson disease	3.333036583989179e-05	8.351427918110758e-05
hypogonadism	3.340571167244303e-05	8.351427918110758e-05
breast cancer	4.6111723066658075e-05	0.0001121636507026818
thyroid carcinoma	4.965752478124495e-05	0.00011760992711347488
deafness	7.732298610928008e-05	0.00017843766025218478
corneal dystrophy	8.694225006945731e-05	0.00019562006265627896
mental retardation	0.0001963376965130264	0.00043098518746761895
ichthyosis	0.00036153323892730864	0.0007747140834156613
dementia	0.0005701702093571497	0.0011662572464123516
skin/hair/eye pigmentation	0.0005701702093571497	0.0011662572464123516
ataxia	0.0012120519296486677	0.0024241038592973355
ectodermal dysplasia	0.0014314931864819092	0.0026292731996606497

ehlers-danlos	0.0014314931864819092	0.0026292731996606497
hypothyroidism	0.0014314931864819092	0.0026292731996606497
thrombophilia	0.0014314931864819092	0.0026292731996606497
hypertension	0.0015215665595955032	0.002738819807271906
anomalies	0.0017241738593789223	0.0028736230989648706
fanconi anemia	0.0017241738593789223	0.0028736230989648706
ovarian cancer	0.0017241738593789223	0.0028736230989648706
rheumatoid arthritis	0.0017241738593789223	0.0028736230989648706
cone-rod dystrophy	0.0018960581428467006	0.0031026405973855102
lung cancer	0.0019320108736484382	0.003105017475506419
orofacial cleft	0.001977196255800001	0.00312188882494737
schizophrenia	0.0026187111595429176	0.004063517316532114
zellweger syndrome	0.004015473640482493	0.0061252987736173615
dystonia	0.004114670322098158	0.006172005483147237
malaria	0.004218584717340146	0.006224141386239559
lateral sclerosis	0.007778921022232993	0.011112744317475705
microphthalmia	0.007778921022232993	0.011112744317475705
osteoporosis	0.009145253170131623	0.012860512270497594
microcephaly	0.018085125140114407	0.02504094250169687
fibrosis	0.019404835294583832	0.02646113903806886
prostate cancer	0.019778485377028875	0.026568114685561178
systemic lupus erythematosus	0.022633736356201938	0.02995641576556139
arrhythmogenic right ventricular dysplasia	0.0357791342807951	0.046001744075307986
melanoma	0.0357791342807951	0.046001744075307986
