# Supplementary material for: Molecular Modelling Hurdle in the Next-Generation Sequencing Era
Source: Int J Mol Sci. 2022 Jun 28;23(13):7176. doi: 10.3390/ijms23137176 (PMC9266691; doi:10.3390/ijms23137176)
Supplement: Supplementary file 1 [file ijms-23-07176-s001.zip › Table_S2.pdf]

Table S2. SnpEFF Impact notation definition.

Impact	Meaning	Example Type	Example Variant
**HIGH**	The variant is assumed to have disruptive impact in the protein	Stop_gain	IGSF·(NM_001007237.3): c.1724G>A/ p.Trp575*
**MODERATE**	Non-disruptive variant that might change protein effectiveness	Missense	DNAH3(NM_001347886.2):c.9191A>G/ p.Asn3064Ser
**LOW**	Variant unlikely to change protein behavior	Synonymous	CDC27(NM_001256.6):c.1458T>C/ p.Asn486Asn
**MODIFIER**	Non-coding variant where there is no evidence of impact	3′-UTR	XPA(NM_000380.4):c.*203C>G
