# Supplementary material for: Molecular Modelling Hurdle in the Next-Generation Sequencing Era
Source: Int J Mol Sci. 2022 Jun 28;23(13):7176. doi: 10.3390/ijms23137176 (PMC9266691; doi:10.3390/ijms23137176)
Supplement: Supplementary file 1 [file ijms-23-07176-s001.zip › Table_S3.pdf]

Table S3. Main MECP2 transcripts description.

Transcript ID	Bp	Protein	UniProt	RefSeq
ENST00000453960	10,343	498aa	P51608-2	NM_001110792.2
ENST00000303391	10,467	486aa	P51608-1	NM_004992.4
